# A new high spatial density temperature dataset in the Grenoble alpine valley (France) for urban heat island investigation and climate services dedicated to municipalities purposes

**DOI:** 10.1016/j.dib.2024.110553

**Published:** 2024-05-25

**Authors:** Xavier Foissard, Sandra Rome, Sylvain Bigot, Emilie Rousset, Anne-Cécile Fouvet

**Affiliations:** a Grenoble Alpes University, Institute of Geoscience and Environment (IGE, UMR 5001), CS 40700 - 38058 Grenoble Cedex 9, France; b Ville d'Echirolles, 1, place des Cinq Fontaines - BP 248, 38433 Echirolles Cedex, France; c Ville de Grenoble, 11, boulevard Jean Pain - CS 91066 - 38021 Grenoble Cedex 1, France

**Keywords:** Thermal sensors, Air temperature, Urban climate, Local climate zone (LCZ), Mountain context, Heatwave, Decision tool mapping

## Abstract

Within the study of the urban heat island (UHI) in Echirolles and Grenoble (France, the eastern part of the alpine arc), two temperature measurement networks have been deployed. The aim is to measure the temperature gradients associated with the UHI in summer. A total of 62 measurement points has been installed in the various neighborhoods on 3-meter-high streetlights, starting in summer 2019. The preliminary classification of the different neighborhood typologies according to ``Local Climate Zone'' guided the choice of location for the temperature sensors. These urban observations respond to a dual challenge: firstly, to observe temperature located in complex topographical situations with valleys, and secondly, to observe the urban climate in neighborhoods where social considerations are important. Municipalities of Echirolles and Grenoble were involved in the investigation. The ADEME-funded (The French Agency for Ecological Transition) CASSANDRE research program analyzes and processes these observations to study the vulnerability of inhabitants to heat waves and more generally to summer heat stress.

Specifications Table

**Table:** 

Subject	Environmental Science: Climatology
Specific subject area	Observing the urban heat island (UHI) in order to map this phenomenon, to contribute to local knowledge for socio-economic issues and town planners
Data format	Raw and analysed (flag)
Type of data	Table
Data collection	The temperature sensors are equipped with a Tinytag Talk 2 data logger (Gemini Data Loggers) with solar RS3 radiation shields (ONSET) protect the probes. The device is mounted on a streetlight 3 m above the ground.
Data source location	Institutions: Ville d'Echirolles and Ville de Grenoble City: Echirolles, Grenoble Country: France Geographical coordinates: cf. data table
Data accessibility	Repository name: Easy Data (French …from French “Data Terra” Research Infrastructure Data identification number: 10.57932/ef63817e-6131-47a9-ab9d-50227fcaac6c Direct URL to data: https://doi.org/10.57932/ef63817e-6131-47a9-ab9d-50227fcaac6c

## 1 Value of the Data

• The data provide observations of the urban heat island (UHI) and when this phenomenon is combined with heat waves. Both cities, Grenoble and Echirolles are located in the French Alps, between three mountain ranges peaking at over 2000 m. Urban observations in a complex topographical context are a valuable contribution to investigating these critical environments.
• The high density of the measurement network is an asset for understanding spatial and temporal variability on the scale of the urban climate.
• These datasets are crucial for research in climatology and environmental studies in urban context.
• These data can be used for applied research, public decision-making, urban planning, urban socio-ecosystems, public health, etc. The data allows the analysis and validation of UHI climate models. It provides ``ground truth''.

## 2 Background

The municipalities of Echirolles and Grenoble need to establish strategies for adapting to climate change, particularly heat waves. In this context, these municipalities have commissioned a study of the urban heat island phenomenon to assess the current situation and develop a method for mitigating this phenomenon. Given the complex topographical context, this study involved setting up a high-density observation network. The measurement network is expected to become a permanent contribution to local climate knowledge for other studies.

The measurement network is focused on urban habitation, while ensuring the representativeness of the urban forms instrumented. The aim is to observe the spatial variability of temperature gradients relative to the UHI, with the intention of map it.

## 3 Data Description

**Fig. 1:**
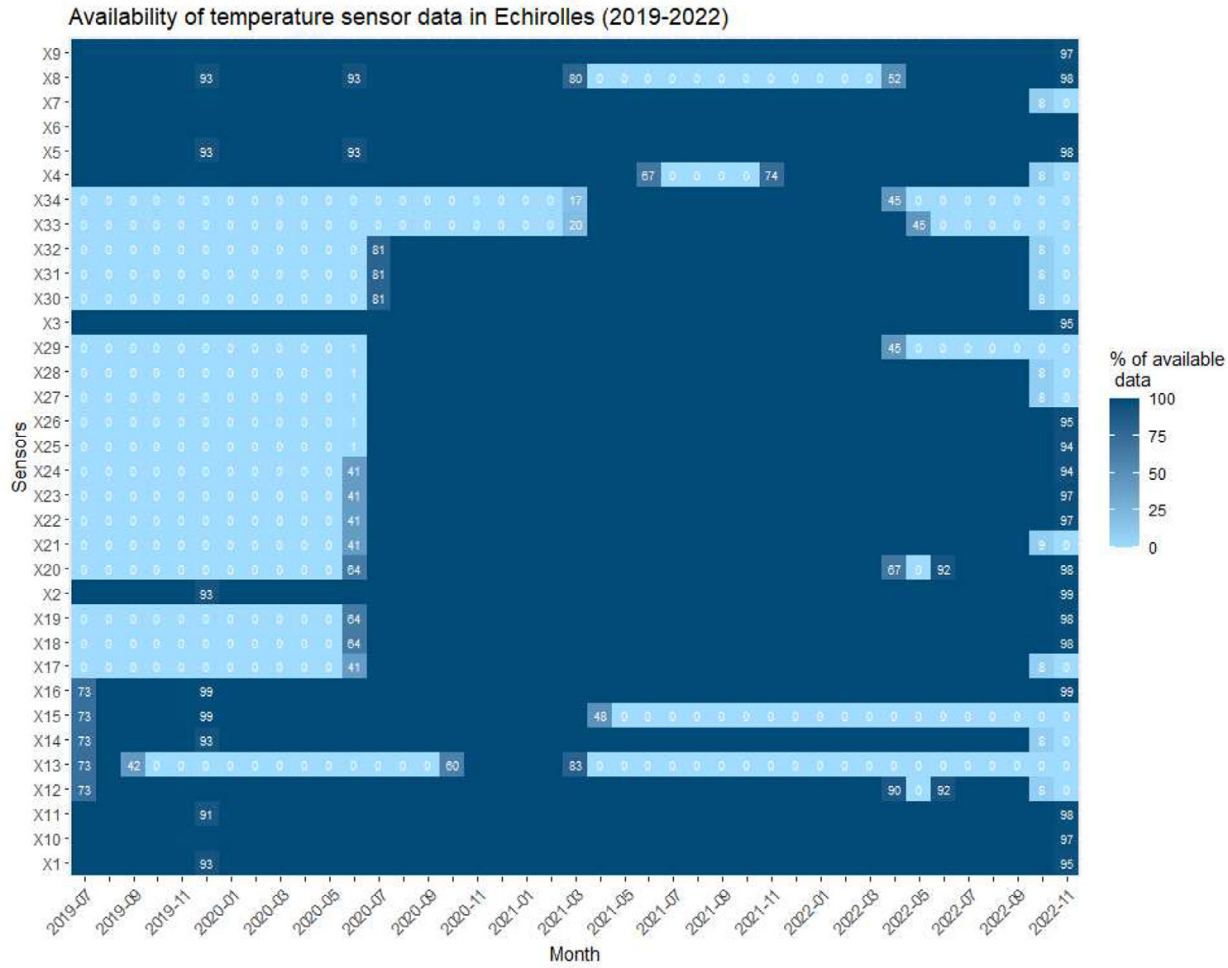
Data availability per sensor temperature in Echirolles.

**Fig. 2:**
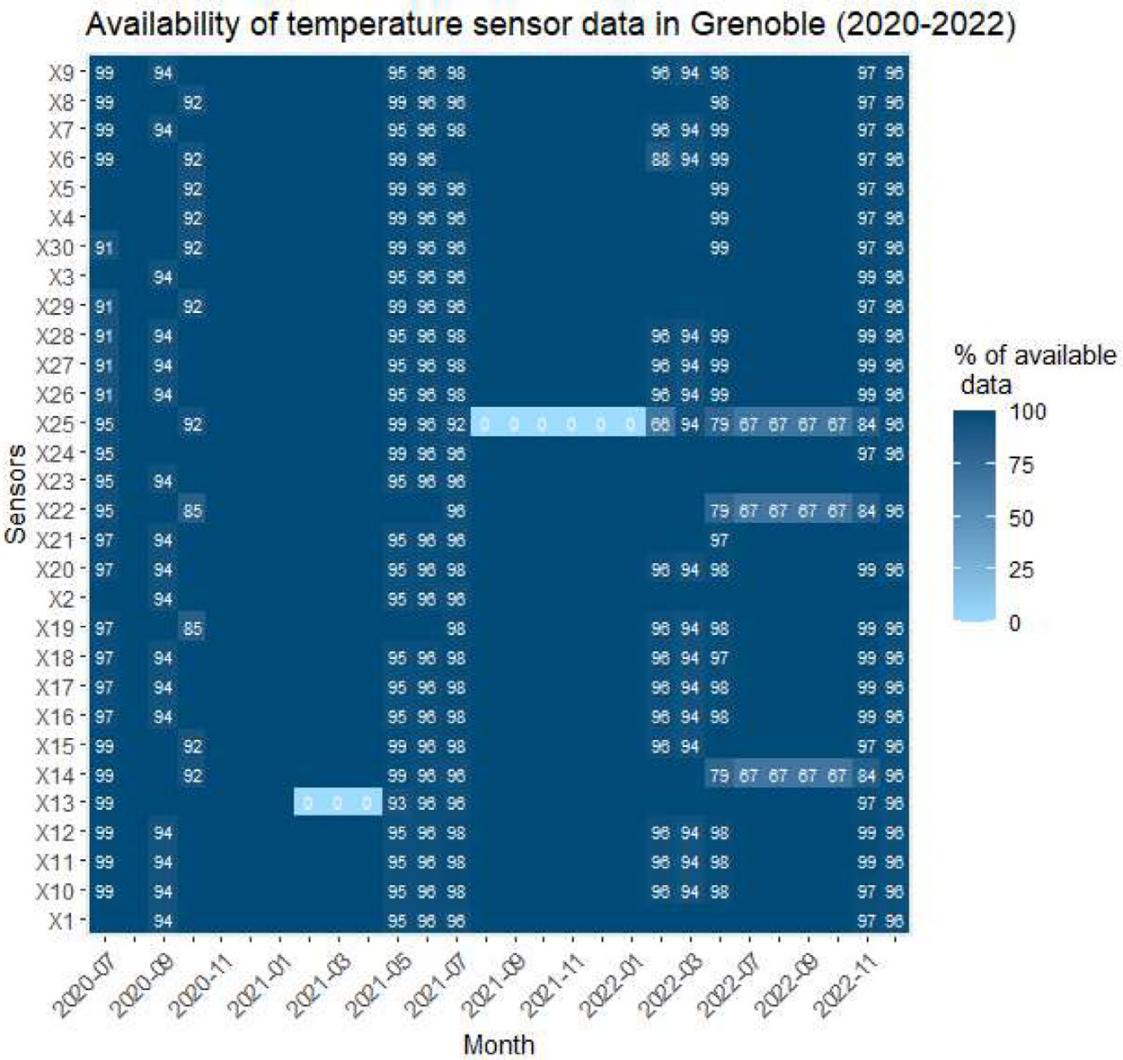
Data availability per sensor temperature in Grenoble.

These data correspond to the temperature recorded by sensors in city. Recordings began in Echirolles (France) in July 2019 with 16 sensors, are completed with 18 additional measuring points in July 2020 and in parallel extended to Grenoble in July 2020 with 30 sensors as described in Table 1 below. These air temperature data provide an observation of the UHI (Urban Heat Island) phenomenon throughout the year, although summer recordings were favored (Figs. 1 and 2).

**Table 1:** Description of the thermal data acquired in Echirolles and Grenoble.

City	Period	Time step	Number of sensors	Unit	Urban description
Echirolles	2019/07/24 to 2019/11/27	20 min	1 6	°C	LCZ #
Echirolles	2019/12/19 to 2022/11/04	1 h	3 2	°C	LCZ #
Grenoble	2020/07/08 to 2022/12/05	1 h	3 0	°C	LCZ #

A total of 62 temperature monitoring points were installed to measure gradients between neighborhoods in various urban context. The temperature data is tabulated for each city. They showed the sensor identification, numbered according to the following format: “X##”. In Echirolles, the first recording campaign in 2019 has begun with a 20-min time step. From December 2019 the hourly time step was used.

Temperature records were formatted as shown in Table 2. The “Date” column corresponds to the Coordinated Universal Time (UTC) date and time in the format: YYYY-MM-DD HH:MM. The “_Max” and “_Min” columns respectively referred to the maximum and minimum temperature recorded during the hour. The heading “_Tem” showed the current measured temperature. The prefix "FLAG" indicated the quality of the data, as described in the LIMITATIONS section of this data paper.

**Table 2:** Data content and format (## = sensor identification number).

Date	X##_Max	X##_Min	X##_Tem	FLAG_X##
*YYYY-MM-DD HH:MM* UTC	Maximum temperature	Minimum temperature	Instantaneous temperature	Quality control

Associated with the table, the measurement points are located in the GIS file. The file contains the identifier “X##” of each point and the type of LCZ (Local Climate Zone) corresponding to the neighborhood within the sensor was installed.

## 4 Experimental Design, Materials and Methods

**Fig. 3:**
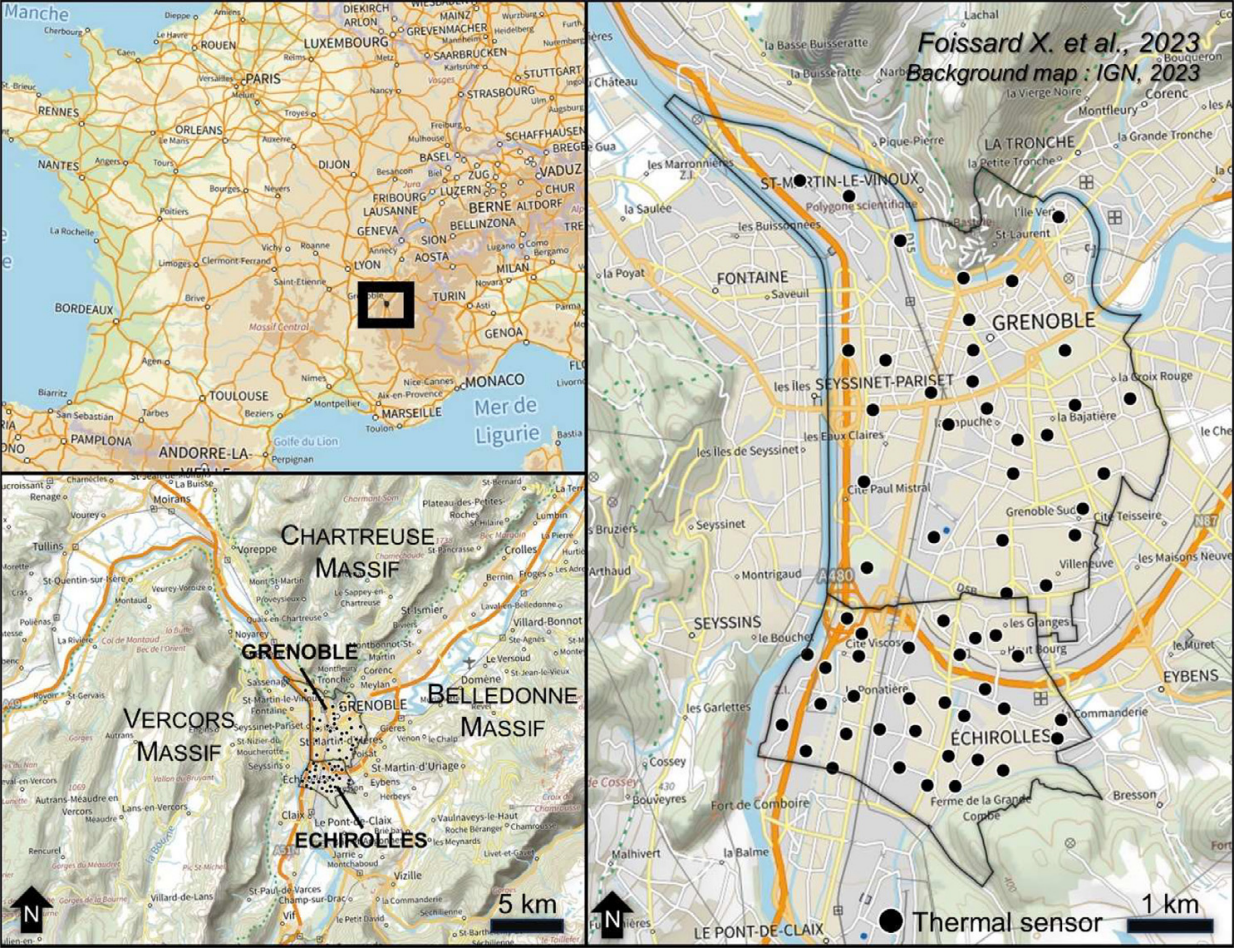
Thermal sensors location in Echirolles and Grenoble (France).

The data produced consist of temperatures measured in two cities: Grenoble (158,000 inhabitants) and Echirolles (37,000 inhabitants). They are respectively the capital of the Isère department and the department's third most populous city. These cities are located to the west of the Alpine arc in French Alps, more precisely at the bottom and intersection of 3 valleys forming a “Y” between the Pre-Alps and the Alps (45°10′N 5°43′E). The surrounding relief reaches altitudes of over 2000 m. The climate of this relatively flat valley floor area is altered oceanic climate (Cfb according to Köppen's classification), with influences from mountain margins [1]. However, the cities are built on a glacial trough (flat-bottomed valley) whose altitude varies very slightly [2]. We can therefore consider that all the sensors are located at an altitude of 220 m (+/−10 m). This specific topographical context generates an altitudinal thermal gradient, varying between valley bottoms and altitudes in south-facing or north-facing slope positions [3] and also generates both valley and mountain breezes. For this reason, the network for monitoring temperatures in the city responds to a need to assess the intensity of the UHI through measurement, and to establish an in-situ reference (Fig. 3).

**Fig. 4:**
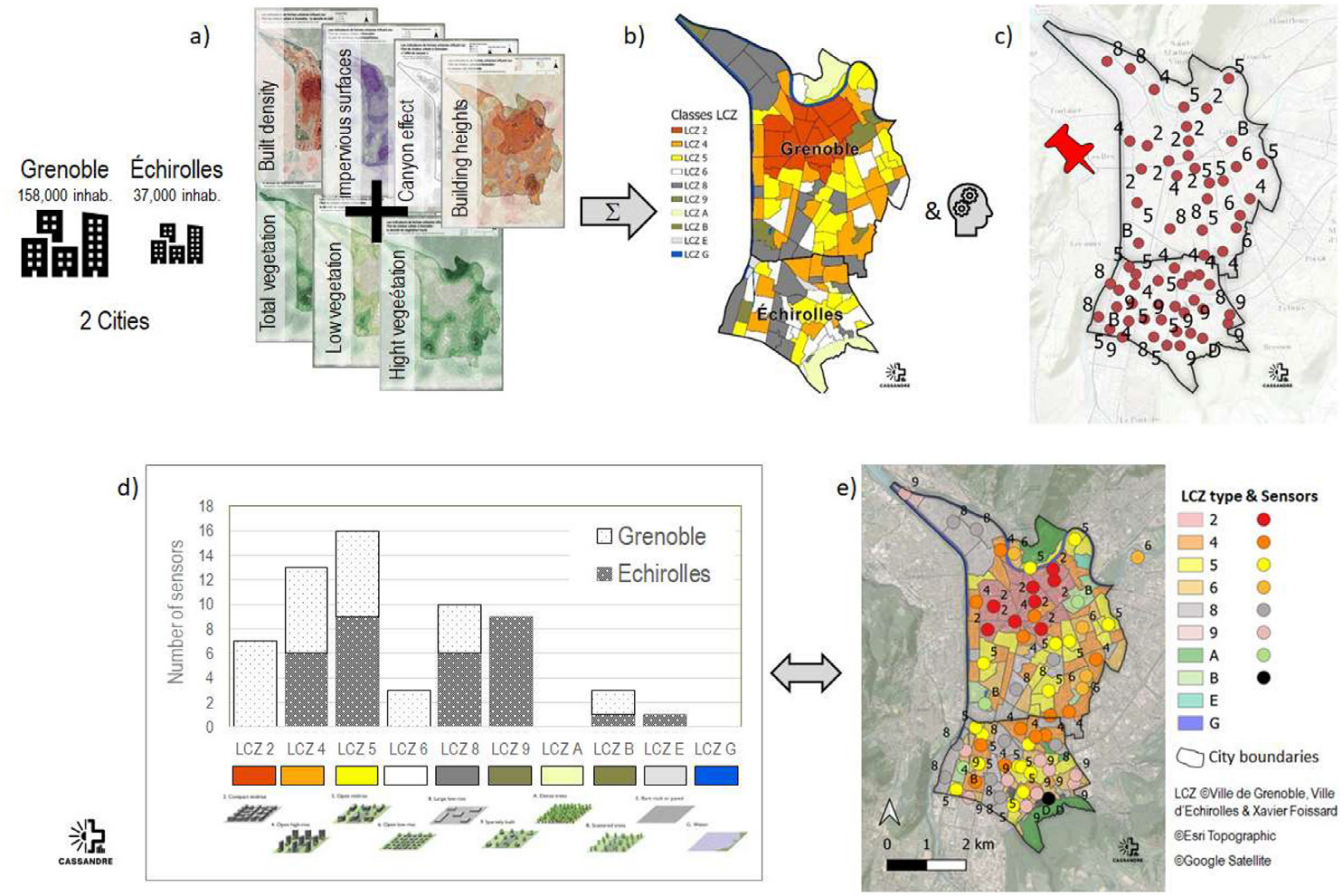
Process leading to define location of the thermal sensors. a) Spatialization of 7 indicators to establish coherent zones; b) LCZ delimitation; c) Location of sensors; d) Number of sensors by LCZ type and by municipality; e) Final map of LCZs and associated thermal sensors.

The choice of location of temperature sensor networks was based on the Local Climate Zone (LCZ) methodology [4]. LCZ can be used to describe neighborhoods on an urban climatic scale. This classification is based on morphological indicators. For the UHI study, this approach ensured that the observation network was representative. The first step was to map urban form indicators relevant to the phenomenon of UHI (cf. Fig. 4). The second step consists of subdividing neighborhoods according to LCZ, based on these spatialized indicators and supervised by town planners. From the LCZ, sensors were installed according to the different morphological configurations of the city, to be representative of the variability of urban typologies while avoiding micro-local effects [5].

Prior to installation, the sensors were tested during a preliminary recording phase on the same site. The sensors were installed about 3 m above the ground on lampposts to avoid any risk of damage. The temperature sensors were equipped with a Tinytag Talk 2 data logger (Gemini Data Loggers) protected by a solar RS3 radiation shield (ONSET). Loggers were powered by lithium batteries and the accuracy was ±0.4 °C. The sensors were placed at the greatest distance from the nearest building to limit micro-local effects. This distance was adapted according to the type of neighborhood. The density of thermal sensors in urban area was 2.4 points/km² in Grenoble and 4.5 points/km² in Echirolles.

## Limitations

Direct sun exposure can lead to over-estimation of the temperature by overheating the sensor in the shelter. Care should be advised when using and interpreting maximum temperatures. Care should be advised when using and interpreting maximum temperatures. UHI observation is not affected by this effect due to its nocturnal period.

A first quality control is proposed by evaluating the data according to three criteria, cf. Table 3.

**Table 3:** Quality control modalities.

Quality control of hourly temperature	Code
“Good”	1
“Suspicious”	2
“Missing”	0

These criteria are defined by the standard deviation where the instantaneous temperature is greater than 4 times the standard deviation is considered ``Suspicious'' data.

## Ethics Statement

The authors have read and follow the ethical requirements for publication in Data in Brief and confirming that the current work does not involve human subjects, animal experiments, or any data collected from social media platforms.

## CRediT authorship contribution statement

**Xavier Foissard:** Methodology, Data curation, Writing – original draft, Visualization. **Sandra Rome:** Writing – original draft, Writing – review & editing, Visualization. **Sylvain Bigot:** Supervision, Writing – review & editing. **Emilie Rousset:** Supervision, Project administration. **Anne-Cécile Fouvet:** Supervision, Project administration.

## Data Availability

A new high spatial density temperature dataset in the Grenoble alpine valley (France) for urban heat island investigation and climate services dedicated to municipalities purposes (Original data) (Easy Data).
